# Reduced Salivary Lactoferrin Levels in Early-Onset Alzheimer's Disease

**DOI:** 10.14336/AD.2023.0819

**Published:** 2024-05-07

**Authors:** Desireé Antequera, Laura Carrero, Marta Gonzalez-Sanchez, José Luis Cantero, Gorka Orive, Cristina Municio, Eva Carro

**Affiliations:** ^1^Neurobiology of Alzheimer's disease Unit, Functional Unit for Research into Chronic Diseases, Instituto de Salud Carlos III, Madrid, Spain; Network Centre for Biomedical Research in Neurodegenerative Diseases (CIBERNED), ISCIII, Spain.; ^2^Group of Neurodegenerative Diseases, Hospital Universitario 12 de Octubre Research Institute (imas12), Madrid, Spain; Network Center for Biomedical Research in Neurodegenerative Diseases (CIBERNED), ISCIII, Spain.; ^3^Laboratory of Functional Neuroscience, Pablo de Olavide University, Seville, Spain; Network Center for Biomedical Research in Neurodegenerative Diseases (CIBERNED), ISCIII, Spain; ^4^Laboratory of Pharmacy and Pharmaceutical Technology, Faculty of Pharmacy, University of the Basque Country/Euskal Herriko Unibertsitatea (UPV/EHU), Vitoria, Spain; Bioaraba, NanoBioCel Research Group, Vitoria-Gasteiz, Spain; Networking Center for Biomedical Research in Bioengineering Biomaterials and Nanomedicine (CIBER-BBN), ISCIII, Spain.

**Dear Editor**,

Decreased salivary lactoferrin levels were reported in patients suffering from mild cognitive impairment (MCI) and AD compared with age-matched healthy subjects [1], indicating a putative link between AD, the immune system, and brain infections [2]. More recently, salivary lactoferrin has been proposed to be useful to discriminate AD from other dementias, as salivary lactoferrin levels are associated with the amyloid-PET imaging profile [3]. Moreover, it has been described that salivary lactoferrin is negatively associated with regional amyloid-β (Aβ) load and poorer memory [4]. However, it was not yet known when lactoferrin levels began to drop in those patients suffering from AD.

AD is often separated according to age, whereby onset prior to age 65 years is considered to be early-onset AD (EOAD), while onset from an age of 65 years is termed late-onset AD (LOAD). Both AD forms follow a similar pathological and biochemical course, but show distinct cognitive features, and no correlation between the core biomarkers and age in AD patients [5]. It is known that age may influence salivary lactoferrin production. A gradual reduction in salivary lactoferrin concentration was observed in subjects from their fourth decade onward [6]. Therefore, we aimed to analyze age-related differences in salivary lactoferrin levels in patients with EOAD and LOAD.

In this study, we performed a cross-sectional sampling from longitudinal cohorts at the 12 de Octubre University Hospital (Madrid, Spain) and Pablo de Olavide University (Sevilla, Spain) of participants with a diagnosis of EOAD and LOAD, establishing diagnostic criteria of dementia due to AD [7]. We required a positive amyloid-PET scan for subjects meeting only “possible AD” (n = 34). Global cognition was assessed using the Mini Mental State Examination (MMSE) [8]. Functional impairment was measured via the Clinical Dementia Rating (CDR) score [9]. Demographic and clinical data of participants are shown in Supplementary Table 1. All study participants provided informed consent, and the study protocols were approved by the Research Ethics Committee of each entity. Saliva samples were collected and processed from all subjects as described previously [1], and lactoferrin levels were determined using the Lf human ELISA (Abcam).

**Figure 1.**
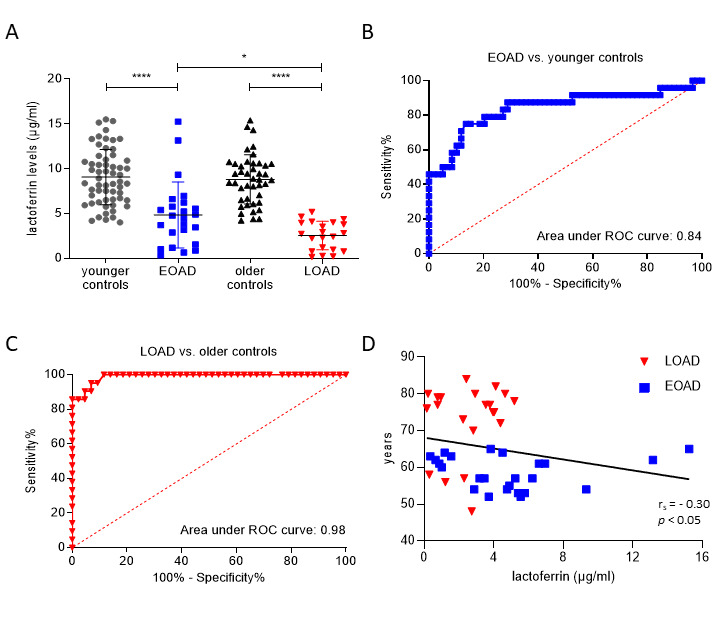
**Salivary lactoferrin levels in control, EOAD, and LOAD groups**. (**A**) Plots for all four-participant groups with median lines added for each group. Values for the mean ± SD are given. Differences between groups were assessed using one-way ANOVA followed by Tukey test (*p < 0.05, ****p < 0.0001). (B, C) ROC curves and their corresponding areas under the curve (AUCs) to differentiate between (B) EOAD patients and younger controls, and (C) LOAD patients and older controls. (**D**) Correlation between age and salivary levels of lactoferrin in AD patients using the Spearman rank-order test. Abbreviations: EOAD, early-onset Alzheimer’s disease; LOAD, late-onset Alzheimer’s disease; ROC, receiver operating characteristic.

Salivary lactoferrin levels were significantly lower in both EOAD and LOAD patients in comparison with age-matched control group (Fig. 1A). Moreover, salivary lactoferrin was significantly decreased in patients with LOAD compared to EOAD. Additionally, no significant differences in levels of salivary lactoferrin were found between the two control groups (Fig. 1A). Then, salivary lactoferrin values were analyzed using logistic regression models. We confirmed that salivary lactoferrin was excellent at separating corresponding controls from patients in LOAD (Supplementary Table 2; Fig. 1B and C). Salivary lactoferrin levels were less accurate at separating young controls from patients with EOAD (Supplementary Table 2; Fig. 1B and C). We also found that lower lactoferrin levels in saliva were associated with older age only in the combined AD group (Fig. 1D).

Here, we found a clear and highly significant decrease in salivary lactoferrin levels in EOAD and LOAD patients compared to corresponding age-matched controls. The most interesting result is that the reduction in salivary levels of lactoferrin was higher in LOAD compared to EOAD. Our present study also supports the potential use of a specific salivary biomarker as a diagnostic tool in the early diagnosis of AD. Early diagnosis of AD is necessary to begin the treatment before the onset of clinical symptoms and to slow down the progression of the disease. Emerging data have highlighted the saliva potential in AD diagnosis offering all advantages to be used in early screening [10].

Our findings agree with the previously known salivary gland dysfunction in AD [11, 12], suggesting that these molecular and functional alterations could be the reason for the reduced levels of lactoferrin in saliva [2]. Notably, a gradual reduction in salivary lactoferrin levels was observed throughout middle age, being statistically significant comparing with subjects from their fourth decade onward [6]. It is well known that aging is associated with immune dysfunction, also called “immunosenescency,” [13]. Thus, our findings support the hypothesis that EOAD is linked with deterioration of systemic immunity probably associated with hypothalamic functional decay, as reflected by a decline in salivary lactoferrin levels.

In conclusion, our data further corroborate and extend previous studies demonstrating that salivary lactoferrin is reduced in EOAD and LOAD patients and supporting that it could be a technically reliable and useful biomarker in AD [14]. Low salivary lactoferrin levels could alter the oral microbiota causing oral dysbiosis, a plausible contributory factor in the development of AD pathophysiology [15]. In the last years, *Infectious Hypothesis* has emerged as an important pathogenic process of AD pathogenesis, closely related to decrease salivary lactoferrin, and with notable connection with *Cholinergic Hypothesis* [16]. Our previous data suggest that the acetylcholine-mediated signaling pathway is impaired in salivary glands in AD, resulting in salivary gland dysfunction and reduced salivary lactoferrin secretion [12]. Therefore, we propose that early reduction in salivary lactoferrin levels may critically alter the oral environmental by controlling infections, enhancing the vulnerability of demented patients [17].

## Supplementary Materials

The Supplementary data can be found online at: www.aginganddisease.org/EN/10.14336/AD.2023.0819.


