# Supplementary material for: Reduced Salivary Lactoferrin Levels in Early-Onset Alzheimer's Disease
Source: Aging Dis. 2024 May 7;15(3):945–7. doi: 10.14336/AD.2023.0819 (PMC11081168; doi:10.14336/AD.2023.0819)
Supplement: Supplementary file 1 [file AD-15-3-945-s.pdf]

SUPPLEMENTARY DATA

Reduced Salivary Lactoferrin Levels in Early-Onset Alzheimer's Disease

Desireé Antequera, Laura Carrero, Marta Gonzalez-Sanchez, José Luis Cantero, Gorka Orive[4], Cristina Municio, Eva Carro

© 2023. Antequera D. et al. Published online at http://www.aginganddisease.org/EN/10.14336/AD.2023.0819

SUPPLEMENTARY DATA

Supplementary Table 1. Demographic and clinical data of the participants.

Characteristic	Control <65 (n = 59)	EOAD (n = 28)	Control >65 (n = 45)	LOAD (n = 25)	*p*-value #
Age, mean (SD), y	58.64 (5.63)	58.85 (4.37)	75.93 (4.38)	77.80 (4.21)	< 0.0001
Female sex, n (%)	44 (74.57)	14 (50.00)	21 (46.66)	14 (56.00)	< 0.05
Disease duration, mean (SD), y	NA	3.30 (2.66)	NA	3.98 (2.05)	ns
MMSE score, mean (SD)	29.27 (1.27)	21.45 (4.72)	28.90 (1.52)	19.42 (5.85)	< 0.0001
CDR, mean (SD)	0	0.97 (0.46)	0	1.40 (0.63)	NA
APOE ε4 carrier, No. (%)	8 (13.60)	16 (57.14)	8 (17.77)	13 (52.00)	< 0.0001
Saliva Lf, μg/ml (SD)	9.07 (3.08)	4.84 (3.68)	8.89 (2.27)	2.92 (1.54)	< 0.0001

Abbreviations: EOAD, early-onset Alzheimer's disease; LOAD, late-onset Alzheimer's disease; MMSE, Mini-Mental State Examination; CDR, Clinical Dementia Rating; Lf, Lactoferrin; NA, not applicable; ns, non-significant. #*p* value indicates statistical difference.

Supplementary Table 2. Sensibility and specificity of salivary lactoferrin.

	Younger control vs. EOAD	Older control vs. LOAD
AUC	0.84	0.98
(95% CI)	(0.73, 0.95)	(0.95, 1)
Sensitivity, %	62.62	83.89
Specificity, %	72.88	90.70
Cut-off, μg/ml	6.65	5.27

Performance of salivary lactoferrin analysed by receiver operating characteristic (ROC) curve. The optimal cut-off point to differentiate patients with AD from healthy controls from each cohort was used to calculate sensibilities and specificities Abbreviations: EOAD, early-onset Alzheimer's disease; LOAD, late-onset Alzheimer's disease; AUC: area under the ROC curve.

© 2023. Antequera D. et al. Published online at http://www.aginganddisease.org/EN/10.14336/AD.2023.0819
